# The Mitochondrial RNA Landscape of *Saccharomyces cerevisiae*

**DOI:** 10.1371/journal.pone.0078105

**Published:** 2013-10-15

**Authors:** Edward M. Turk, Vaijayanti Das, Ryan D. Seibert, Erik D. Andrulis

**Affiliations:** 1 Department of Molecular Biology and Microbiology, Case Western Reserve University School of Medicine, Cleveland, Ohio, United States of America; 2 Science Department, Gilmour Academy, Gates Mills, Ohio, United States of America; Univ. of Edinburgh, United Kingdom

## Abstract

Mitochondria are essential organelles that harbor a reduced genome, and expression of that genome requires regulated metabolism of its transcriptome by nuclear-encoded proteins. Despite extensive investigation, a comprehensive map of the yeast mitochondrial transcriptome has not been developed and all of the RNA-metabolizing proteins have not been identified, both of which are prerequisites to elucidating the basic RNA biology of mitochondria. Here, we present a mitochondrial transcriptome map of the yeast S288C reference strain. Using RNAseq and bioinformatics, we show the expression level of all transcripts, revise all promoter, origin of replication, and tRNA annotations, and demonstrate for the first time the existence of alternative splicing, mirror RNAs, and a novel RNA processing site in yeast mitochondria. The transcriptome map has revealed new aspects of mitochondrial RNA biology and we expect it will serve as a valuable resource. As a complement to the map, we present our compilation of all known yeast nuclear-encoded ribonucleases (RNases), and a screen of this dataset for those that are imported into mitochondria. We sought to identify RNases that are refractory to recovery in traditional mitochondrial screens due to an essential function or eclipsed accumulation in another cellular compartment. Using this *in silico* approach, the essential RNase of the nuclear and cytoplasmic exosome, Dis3p, emerges as a strong candidate. Bioinformatics and *in vivo* analyses show that Dis3p has a conserved and functional mitochondrial-targeting signal (MTS). A clean and marker-less chromosomal deletion of the Dis3p MTS results in a defect in the decay of intron and mirror RNAs, thus revealing a role for Dis3p in mitochondrial RNA decay.

## Introduction

Mitochondrial inheritance was first discovered in 1949 in the yeast, *Saccharomyces cerevisiae*, by the group of Ephrussi and Slonimski[1] (see 2 for a review). Yeast has since remained a model organism for the investigation of mitochondrial genetics. In 1996 the sequence of the16 nuclear chromosomes of wild-type yeast S288C[3,4] was released, and two years later the complete sequence of the mitochondrial genome of the same strain was published[5]. The availability of a sequenced genome, and the subsequent creation of large-scale isogenic collections, such as the deletion of every open reading frame (ORF)[6], has made S288C the cornerstone of contemporary yeast genetics. Recently, the entire nuclear transcriptome of S288C was presented in the seminal RNAseq publication[7]; however, the mitochondrial transcriptome has not been sequenced.

The mitochondrial genome of S288C contains 35 genes required for oxidative phosphorylation. Polypeptides are encoded by 8 of those genes, which includes a ribosomal subunit (*VAR1*), cytochrome oxidase subunits I, II and III (*COX1*, *COX2*, *COX3*), apocytochrome b (*COB*), and three ATP synthase subunits (*ATP6*, *APT8*, *ATP9*). The other 27 genes encode RNAs required for translation of the 8 polypeptides, and they include 24 tRNAs, two ribosomal RNAs (*21S rRNA* and *15S rRNA*) and the RNA subunit of RNase P (*RPM1*). The *21S rRNA* gene contains an intron, *COB* contains five, and *COX1* contains seven introns. All are group I or group II introns, which are mobile genetic elements that undergo protein-assisted, autocatalytic, RNA splicing[8,9]. There are also 11 ORFs that show sequence similarity to homing endonuclease genes (HEG)[10]. These ORFs have been shown in some cases to encode active homing endonuclease enzymes, and in other cases the protein appears to have lost DNase activity, or has gained maturase (intron splicing), or reverse transcriptase activity. Ten of the “HEG-related” ORFs are contained within group introns. The exception is *Q0255*, which is a HEG-related ORF that overlaps the 3’ end of the *COX2* ORF, and is not associated with an intron.

Sequencing of the human mitochondrial transcriptome provided a recent example of the importance of post-transcriptional mechanisms to differential gene expression by showing differential abundance for mRNAs that originate from the same polycistronic transcript[11]. An intensely studied mitochondrial post-transcriptional mechanism is RNA decay of the yeast and human degradosome[12]. The degradosome was first discovered in yeast as a Suv3p-Dss1p complex with RNA helicase and 3’-5’ RNA exonuclease activity[13]. The recently discovered human degradosome is analogous to the yeast degradosome, but consists of a hSuv3-PNPase complex[14]. Suv3p is a conserved RNA helicase[15,16], while Dss1p and PNPase are the only known mitochondrial RNA decay ribonucleases. 

Many RNA decay complexes contain 3’-5’ exoribonucleases, such as Rrp6p in the nuclear exosome, Dis3p of the nuclear and cytosolic exosome, PNPase of the bacterial degradosome, and the aforementioned PNPase and Dss1p of their respective human and yeast mitochondrial degradosome. PNPase is a phosphorolytic RNase PH-like exoribonuclease[17], while Rrp6p, Dss1p, and Dis3p are hydrolytic exoribonucleases of the RNase D[18], RNR (RNase II-like)[19,20], and RNase II/R family[21,22], respectively. Thus mode of action and homology are less important than the functional analogy of RNA decay in the 3’ to 5’ direction. Dss1p plays a significant role in mRNA surveillance and intron turnover in yeast mitochondria, but does not appear to be the only decay pathway in this system, because some RNAs have no change in level when *DSS1* is genetically perturbed[13]. In addition to Dss1p, Dis3p and Rrp6p, the yeast nuclear genome encodes many other 3’-5’ exoribonucleases, but does not encode PNPase[23]. Thus it appears that a novel yeast mitochondrial ribonuclease remains to be identified.

Here we report an RNAseq analysis of highly purified mitochondria from the yeast S288C reference strain. This analysis advances our understanding of the identity, boundaries, splicing, cleavage, and quantities of mitochondrial RNAs. In addition, we report a comprehensive *in silico* search for yeast RNases and subsequent experiments that show Dis3p to be a mitochondrial protein involved in non-coding RNA (ncRNA) decay.

## Materials and Methods

### Yeast Strains and Growth Conditions

Yeast wild-type strain, BY4741 (ATCC #201388 http://www.atcc.org/), was obtained from Open Biosystems (https://www.openbiosystems.com) (Cat. #: YSC1048). BY4741 [MATa, *his3-Δ1*, *leu2-Δ0*, *met15-Δ0*, *ura3-Δ0*] is a haploid derivative of S288C[24]. All other yeast strains used in this reports are derivatives of BY4741 (Table S1 in File S1).

Yeast cultures were initiated from frozen stocks by inoculation into 5 mL of YPD (1% yeast extract, 2% peptone, 2% dextrose) in a 14 mL round-bottom culture tube, followed by overnight incubation at 30°C and 245 rpm shaking. Over-night cultures were used to inoculate the experimental culture (1 L of YPD in a 4 L Belco baffle-bottom flask) to an OD600 of 0.5, followed by incubation at 30°C and 245 rpm for two days. 

### RNAseq

Four independent yeast cultures were grown for RNAseq analysis (two BY4741 and two *dis3Δmts*). Highly purified yeast mitochondria were isolated from ~10 g wet-weight of each culture according to published protocols[25]. The sucrose-gradient purified mitochondria were treated with cyanase, a highly active and non-specific RNase/DNase, to mitigate cytoplasmic nucleic acid contamination. Mitochondria were re-purified and lysed in QIAzol from Qiagen. Total mitochondrial RNA was purified using the microRNeasy kit from Qiagen, with on column DNase treatment to remove mitochondrial genomic DNA. 

All RNAseq (random sequencing of whole transcriptome) steps were performed by the genomic core facility at the Huntsman Cancer Institute including validation of RNA quality, quantity and purity; library preparation using the Illumina TruSeq Directional RNA protocol with fragmentation to reduce insert size to 30-300 bases, no DSN treatment, no polyA selection, no ribosomal or tRNA subtraction, and no size selection; and 101 cycle directional paired-end sequencing on an Illumina HiSeq2000 platform. Resulting sequence data was processed using Illumina's CASAVA software, version 1.7.0. to retain only high-quality reads, which were mapped with Bowtie using the Galaxy main instance[26-28]. Avadis NGS software was used to visualize and analyze mapped reads. Expression was measured in RPKM: # of reads mapped to a gene / (size of the transcript in kb) (total # of mappable reads in millions). P-values were calculated using the DESeq statistical method, which is based on the negative binomial distribution with variance and mean linked by local regression; n=2[29]. When comparing the two *DIS3* replicates to each other, or comparing the two *dis3∆mts* replicates to each other, there are no significant differences when the same gene is compared between two replicates.

Due to paired-end sequencing, two RNAseq datasets result from each RNA sample, a dataset of reads in the 5’-3’ direction and a dataset in the 3’-5’ direction, for a total of 8 datasets. Only the 5’-3’ datasets were used for this study to avoid the possibility of double-counting the expression of bases from inserts shorter than 202 bp. The 8 datasets have been deposited at the NCBI short read archive with the following accession numbers (5’-3’ dataset; 3’-5’ dataset). WT experiment-GCDA5 (SRR900222; SRR900223), WT experiment-GCDA8 (SRR900186; SRR900220), mutant experiment-N29dis3A5 (SRR900088; SRR900137), mutant experiment-N29dis3A8 (SRR900251; SRR900255).

### RT-PCR and Sequencing of Alternative-Spliced aI5β

Gene-specific primers used to generate cDNA, amplify the alternative-spliced product, and sequence the resulting PCR product are described in Table S2 in File S1. SuperScript^TM^ III reverse transcriptase (Invitrogen) and the recommended protocol were used to synthesize cDNA from 50 ng of mitochondrial RNA from yeast strain BY4741, except the RNA was incubated with primers and dNTPs at 70°C for 5 min. Five units of RNase H (New England Biolabs) were used to digest the RNA complementary to the cDNA. The cDNA was then diluted 1:5 and 2 μL was used as a template for PCR. Immolase hot start DNA polymerase (Bioline) was used to amplify the spliced product with the following reaction conditions: 1x immobuffer, 3 mM MgCl_2_, 0.3 mM dNTP, 1 μM of forward and reverse primer, and 2.5 units of polymerase in a 50 μL reaction volume. The reaction was denatured at 95°C for 7 min, followed by 35 cycles of 95°C for 30s / 50°C for 30s / 72°C for 30s, and then 4°C hold. Reaction products were separated on a 1% agarose, 1X TAE horizontal gel, detected using ethidium bromide staining, and visualized with GeneSnap software (Syngene). A single 444 bp band was seen, which is the sized predicted for amplification of alternative-spliced aI5β. The PCR product was purified using the Qiaquick PCR purification kit (Qiagen), and then sequenced in both directions using the forward and reverse PCR primers, which resulted in 2-fold coverage over the splice junction. 

### Molecular Modeling and Mitochondrial Targeting Prediction

Full-length Dis3p and Arg8p were modeled using the I-TASSER web accessible program[30,31]. Prediction of mitochondrial targeting likelihood and targeting sequences was performed using the MitoProtII-v1.101 web-accessible program[32]. The amino acid sequences used for both web programs were obtained from SGD.

### Helical Wheel Projections

The N-terminus of Dis3p and Arg8p were assayed for the ability to form an amphipathic alpha helix using the Helical Wheel Projections program Version: Id: wheel.pl,v 1.4 2009-10-20 21:23:36 don Exp, created by Don Armstrong and Raphael Zidovetzki at the University of California Irvine. Web address: http://rzlab.ucr.edu/scripts/wheel/wheel.cgi. Dis3p has a 17 amino acid amphipathic alpha helix starting with lysine 11 (K11). Arg8p has a 15 amino acid amphipathic alpha helix starting with leucine 6 (L6). Hydrophilic residues are circles, hydrophobic residues are diamonds, potentially negatively charged are triangles, and potentially positively charged are pentagons. The most hydrophobic residue is green, and the amount of green is decreasing proportionally to the hydrophobicity, with zero hydrophobicity coded as yellow. Hydrophilic residues are coded red with pure red being the most hydrophilic (uncharged) residue, and the amount of red decreasing proportionally to the hydrophilicity. The potentially charged residues are light blue.

### Functional Assay for Mitochondrial Matrix Localization

The *ARG8* gene was cloned from yeast strain BY4741 into the low-copy pRS415 yeast shuttle vector[33]. To facilitate manipulation of the MTS, the 3xFLAG coding sequence was inserted between the MTS and the rest of the ORF to generate pMTS_ARG8_-ARG8. We also engineered a plasmid that contained no MTS coding sequence (pMTS_∆_-ARG8), or contained the MTS from *DIS3* in place of the native MTS (pMTS_DIS3_-ARG8). Plasmids were transformed into an *ARG8*-deletion strain (*arg8-∆0*) that is isogenic to BY4741. Growth on arginine-free medium, which was scored after 3 days at 30°C, requires localization of Arg8p to the mitochondrial matrix. Plasmids and primers used in this experiment are described in Table S3 in File S1 and Table S4 in File S1 respectively. 

### Drosophila Cell Analysis

S2 cell culturing, immunofluorescence, and fractionation were performed as described[34]. Primers used in fly experiments are described in Table S5 in File S1.

### Alignment

The complete 1001 amino acid sequence of *S. cerevisiae* Dis3p/Rrp44p was obtained from SGD and used as a blastp query (refseq_protein, fungi/metazoa, protein-protein, word size 3, BLOSUM62, existence 11, extension 1). The COBALT program was used to perform a multiple sequence alignment of the first 50 sequences.

### Deletion of the DIS3 MTS

A novel homologous recombination strategy was used to insert the *CUP1* promoter between the *DIS3* promoter and ORF. This homologous recombination event simultaneously deleted the MTS (codons 2-29) and maintained expression of *DIS3*. The *CUP1*-containing cassette was then removed by a second homologous recombination event, resulting in a clean and marker-less deletion of the MTS to make strain *dis3Δmts*. Primers used for this experiment are described in Table S5 in File S1.

### RT-qPCR

Gene-specific primers used to generate cDNA and amplify the target are described in Table S6 in File S1. The Bio-Rad iScript SYBR green kit 170-8893 and a Bio-Rad MyiQ2 machine was used for all RT-qPCR experiments. The manufacturer recommended cycle parameters and reaction conditions were followed exactly, including the use of 10 ng of RNA per 50 μL reaction and a final concentration of 300 nM for each primer. For each RNA investigated, RT-qPCR was performed 3 times for each of the 4 RNA samples (2 independent WT RNA samples and 2 independent *dis3Δmts* RNA samples) for a total of 12 experimental reactions plus a no-RT control for each individual RNA analyzed. In each case the resulting Ct values of the 6 WT reactions were compared to the 6 *dis3Δmts* reactions P-values were calculated using the Student’s t-test function built into Microsoft Excel (two tails, two samples equal).

## Results

### Yeast Mitochondrial Transcriptome

To obtain sufficient quantities of total mitochondrial RNA for RNAseq analysis, we grew two independent cultures of yeast BY4741, a derivative of S288C, for two days in rich media. Within the fist day the cells completed the diauxic shift from fermentative growth on dextrose to aerobic growth on ethanol. During fermentative growth the cells divide rapidly and provide the abundant number of fresh cells required for mitochondrial isolation. Aerobic growth during the second day results in slow growing but mitochondrial-active cells. Highly purified mitochondrial RNA from each culture was subjected one time to Illumina Hiseq2000 deep sequencing, which resulted in approximately 9 million mapped reads for each RNA sample (~18 million total reads).

**Figure 1:**
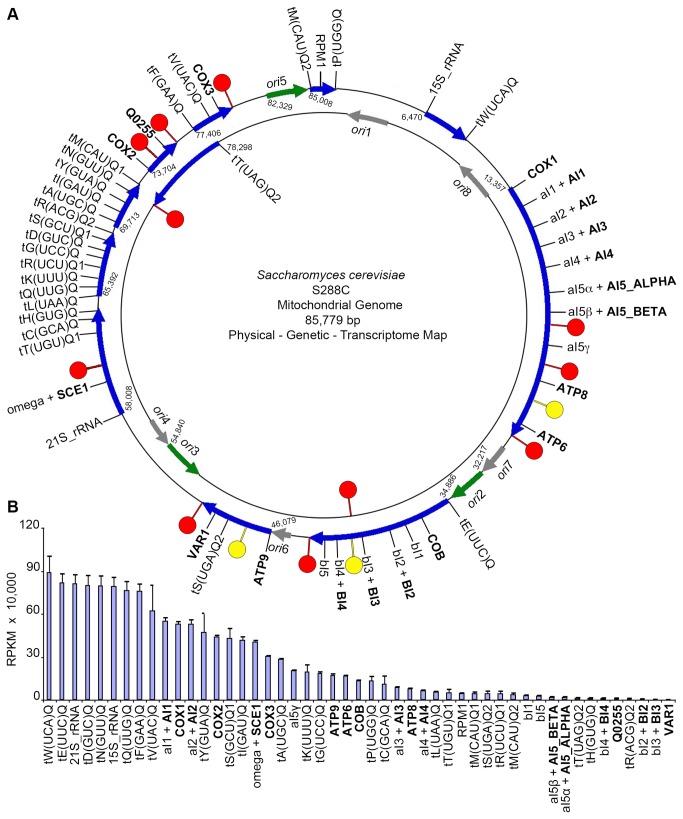
The transcriptome of yeast S288C mitochondria. (A) Physical, genetic, and transcriptome map of the yeast S288C mitochondrial genome. The positive strand is on the outside, and the negative is inside. The active *ori* (green arrows), inactive *ori* (grey arrows), primary transcripts (blue arrows), dodecamers (red lollipops), and deviant dodecamers (yellow lollipops) are modeled. The location of each gene is depicted using the systematic name, protein-coding genes are uppercase/bold, and the transcription start sites are numbered. To mark genes within introns, the intron is lowercase/first, “+”, and then the systematic name of the gene (B) The steady-state RNA abundance of all mitochondrial-encoded genes and introns measured in RPKM.

To create a reference for mapping RNAseq data, we built a model of the S288C mitochondrial genome using DNA sequence information from the Saccharomyces Genome Database (SGD; www.yeastgenome.org), the public repository for this organism [35]. We annotated the genome using information from the SGD genome browser[36] and the primary literature[37]. Most of the experimental data in the literature is based on yeast strains D273-10B, KL14-A4, or W303, which were traditionally used for mitochondrial studies due to good respiratory growth, short mitochondrial genome, or availability, but which are not isogenic to S288C[24,38-40]. We therefore built a parsimonious map of the S288C mitochondrial transcriptome as a working model to be repeatedly tested and refined (Figure 1A).

Mapping the RNAseq reads to our mitochondrial genomic annotation demonstrated clear differential expression of the 35 genes required for oxidative phosphorylation, the group introns, and the HEG-related ORFs (Figure 1B). Notably, there is also differential expression of genes that are on the same primary transcript. For example, the cluster of seven tRNAs on primary transcript starting at nucleotide 65,392 shows nearly 90-fold differential expression between the tRNA with the greatest RPKM (tRNA-Asp) and the tRNA with the least RPKM (tRNA-Arg). The order of genes on the primary transcript is related to the relative abundance in some cases. This is seen by the fact that the *ATP9* gene is followed by tRNA-Ser and then *VAR1* on the same transcript, and the order of abundance is also 5’ to 3’. These results are similar to the results obtained from sequencing the human mitochondrial transcriptome in that co-transcribed RNAs have differential abundance, which points to the importance of attenuated transcription between genes or post-transcriptional RNA processing/decay to provide differential gene expression[11,41].

Sequencing the mitochondrial transcriptome allowed us to determine for the first time the exact 3’ end of all 24 tRNAs. This is made possible by the presence of the non-encoded 3’ terminal CCA that is added post-transcriptionally to all tRNAs. Detection of the CCA addition site also allowed nucleotide-level resolution of the 5’ end of all tRNAs based on tRNA-structure prediction (Table 1). It is notable that the tRNAscan-SE algorithm[42,43] correctly identified 20 of the tRNAs—while primary reports collectively identified all 24—but correctly identified only 2 of the tRNA boundaries[5] (Table 1).

**Table 1:** Yeast mitochondrial-encoded tRNA.

**Systematic Name**	**tRNA Isotype**	**Strand**	**RNAseq tRNA Bounds**		**Mispredicted tRNA Bounds**		**tRNAscan-SE**
			5’	3’	5’	3’	
tP(UGG)Q	Pro	+	731	802			Not Predicted
tW(UCA)Q	Trp	+	9,374	9,444		9,447	1
tE(UUC)Q	Glu	+	35,373	35,444		35,447	2
tS(UGA)Q2	Ser	+	48,201	48,287		48,290	3
tT(UGU)Q1	Thr	+	63,862	63,934		63,937	4
tC(GCA)Q	Cys	+	64,415	64,487		64,490	Not Predicted
tH(GUG)Q	His	+	64,597	64,667	64,596	64,670	5
tL(UAA)Q	Leu	+	66,095	66,176		66,179	6
tQ(UUG)Q	Gln	+	66,210	66,282		66,285	7
tK(UUU)Q	Lys	+	67,061	67,132		67,134	8
tR(UCU)Q1	Arg	+	67,309	67,381			9
tG(UCC)Q	Gly	+	67,468	67,539		67,542	10
tD(GUC)Q	Asp	+	68,322	68,393		68,396	11
tS(GCU)Q1	Ser	+	69,203	69,285		69,288	Not Predicted
tR(ACG)Q2	Arg	+	69,289	69,359		69,362	12
tA(UGC)Q	Ala	+	69,846	69,918		69,921	13
tI(GAU)Q	Ile	+	70,162	70,234		70,237	14
tY(GUA)Q	Tyr	+	70,824	70,908		70,907	Not Predicted
tN(GUU)Q	Asn	+	71,433	71,504		71,503	15
tM(CAU)Q1	Met	+	72,632	72,705	72,630		16
tF(GAA)Q	Phe	+	77,431	77,502		77,505	17
tT(UAG)Q2	Thr	Negative	78,162	78,091		78,089	20
tV(UAC)Q	Val	+	78,533	78,605		78,608	18
tM(CAU)Q2	fMet	+	85,035	85,107		85,112	19

### Promoters and Origin of Replication

To annotate the 5’ end of primary transcripts we analyzed the mitochondrial genomic sequence for the presence of the presumed 9-base yeast mitochondrial promoter, referred to as nonanucleotide[44,45]. We found that 11 promoters can account for expression of all mitochondrial genes and three promoters are associated with origins of replication (Table 2). Five other canonical nonanucleotide sequences also exist but they are not uniquely associated with a transcript (Table 3). 

**Table 2:** Yeast S288C mitochondrial promoters.

**Primary Transcripts**	**Promoter Nucleotides Not Part of the Nonanucleotide (Non-Template Strand)**																			
15s_rRNA, Trp	T	A	T	T										A	T	A	A	A	T	A
COX1, ATP8, ATP6	A	T	T	G										A	T	A	G	A	T	A
*ori2*	A	A	A	T										A	T	A	A	A	T	T
Glu, COB	T	A	T	T										A	T	A	T	A	T	A
ATP9, Ser2, VAR1	A	T	T	A										A	T	A	T	A	T	A
*ori3*	A	G	A	T										A	T	A	G	G	G	G
21S_rRNA, Thr, Cys, His	A	T	A	T										G	T	A	A	A	A	A
Leu, Gln, Lys, Arg, Gly, Asp, Ser, Arg2	T	G	T	T										A	T	A	A	T	A	T
*ori5*	A	A	A	T										A	T	A	G	G	G	G
Ala, Ile, Tyr, Asn, Met	T	T	T	A	T									A	T	A	A	T	A	T
Phe, Val, COX3	T	A	T	A	T									A	T	A	A	T	A	A
Thr2	A	T	T	T	T									G	T	A	T	A	T	A
fMet, RPM1, Pro	G	A	T	T	T									A	T	A	T	A	A	T
COX2, Q0255	T	T	A	A	T			A						G	T	A	T	T	A	A
**Nona-nucleotide**					A	T	A	T	A	A	G	T	A							
**Consensus Promoter**	D	D	W	D	W	T	A	W	A	A	G	T	A	R	T	A	D	D	D	D
**Position**	-12	-11	-10	-9	-8	-7	-6	-5	-4	-3	-2	-1	+1	+2	+3	+4	+5	+6	+7	+8

+ 1 = transcription start site; D = A, T, G (not C); W = A, T (Weak); R = A, G (puRine)

**Table 3:** Nonanucleotides not associated with a consensus promoter.

**Position of Promoter in Genome**	**Nucleotide Deviations From Consensus Promoter (Non-Template Strand)**																			
4810-4829														T	C	C				
33825-33844			C											T						
44443-444462														T	A	C				
46145-46164														T	A	T				
85010-85029														T						
**Nona-nucleotide**					A	T	A	T	A	A	G	T	A							
**Consensus Promoter**	D	D	W	D	W	T	A	W	A	A	G	T	A	R	T	A	D	D	D	D
**Position**	-12	-11	-10	-9	-8	-7	-6	-5	-4	-3	-2	-1	+1	+2	+3	+4	+5	+6	+7	+8

+ 1 = transcription start site; D = A, T, G (not C); W = A, T (Weak); R = A, G (puRine)

The yeast mitochondrial RNA polymerase is of phage T7 origin[46], but the nonanucleotide is considerably smaller than the 23-base phage T7 consensus promoter[47]. We compared the sequences surrounding the minimal set of 14 promoters and derived a 20-base consensus sequence that is not found elsewhere in the mitochondrial genome. The five nonanucleotides that are not uniquely associated with a transcript also do not conform to the 20-base consensus promoter (Table 3). Thus, we conclude that phage T7 and native yeast mitochondrial promoter size is closely conserved, and the minimal set of promoters may also be the full-set.

**Figure 2:**
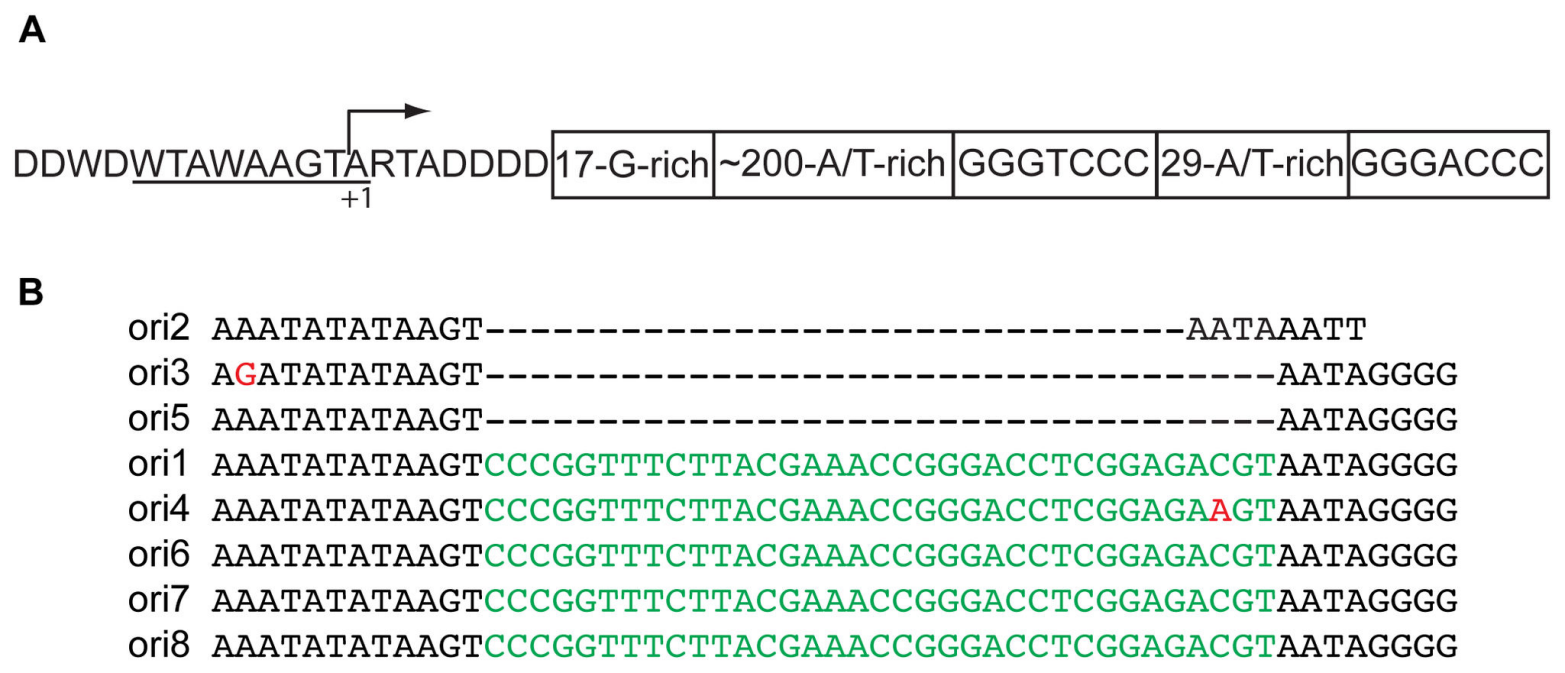
The *ori* of yeast S288C mitochondria. (A) Model based on the presumably active *ori2-3-5*, depicting the non-template strand. The structure consists of a 20-bp promoter (invariable length), followed by a 17-bp G-rich sequence (invariable length and sequence), then an A/T-rich spacer of ~200 bp, followed by two complementary 7-bp sequences (invariable length and sequence). The 7-bp sequences are separated by a 29-bp A/T-rich sequence (invariable length). (B) Alignment of the 20-bp promoters of the potentially active (2-3-5) and inactive (1-4-6-7-8) *ori*. Single nucleotide differences for *ori3* and *4* are highlighted in red, and GC-elements that disrupt the promoter are in green.

Eight yeast mitochondrial origins of replication (*ori*) have been proposed, although not all of the *ori* are active, and those that are active are strain dependent[48]. Our bioinformatic analysis of the S288C mitochondrial DNA sequence shows that 3 *ori* are immediately downstream of the 20-base promoter and conform to a 5-part consensus sequence (Figure 2A). Our RNAseq analysis shows that a contiguous transcript of greater than 350 nucleotides exists for each of the three presumably active *ori*, whereas the other 5 *ori* appear to be disabled by insertions within the promoter, and have few RNAseq reads associated with them. Each of the 5 presumably inactive *ori* contains a 35 bp insert at the identical site within the promoter (Figure 2B).

### Alternative Splicing, Start and Stop Codons

**Figure 3:**
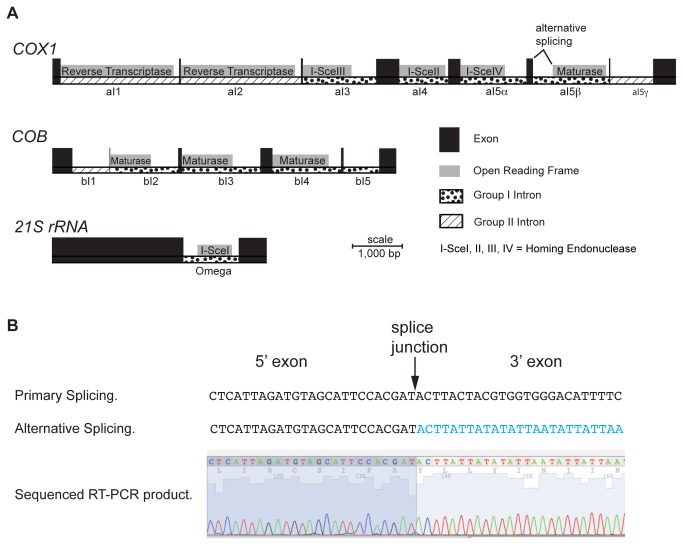
Alternative splicing of aI5β. (A) Model of the 13 introns of yeast S288C mitochondria, and 10 HEG-related ORFs contained within them. The first five introns of *COX1*, and three *COB* introns, contain ORFs that are in-frame with their upstream exon. The ORF within the omega intron of *21S* *rRNA* encodes an ATG at the 5’ end and a dodecamer at the 3’ end. The sixth intron of *COX1*, aI5β, contains an intron that is not in-frame with the upstream exon and does not encode an ATG at the 5’ end. (B) Electropherogram of the sequenced RT-PCR product derived from alternatively spliced aI5β. For comparison, the sequence derived from primary splicing or alternative splicing is depicted above the electropherogram. In both cases the 5’ exon is the sixth *COX1* exon. The 3’ exon is the seventh *COX1* exon in the case of primary splicing and the ORF within the aI5β intron in the case of alternative splicing.

**Figure 4:**
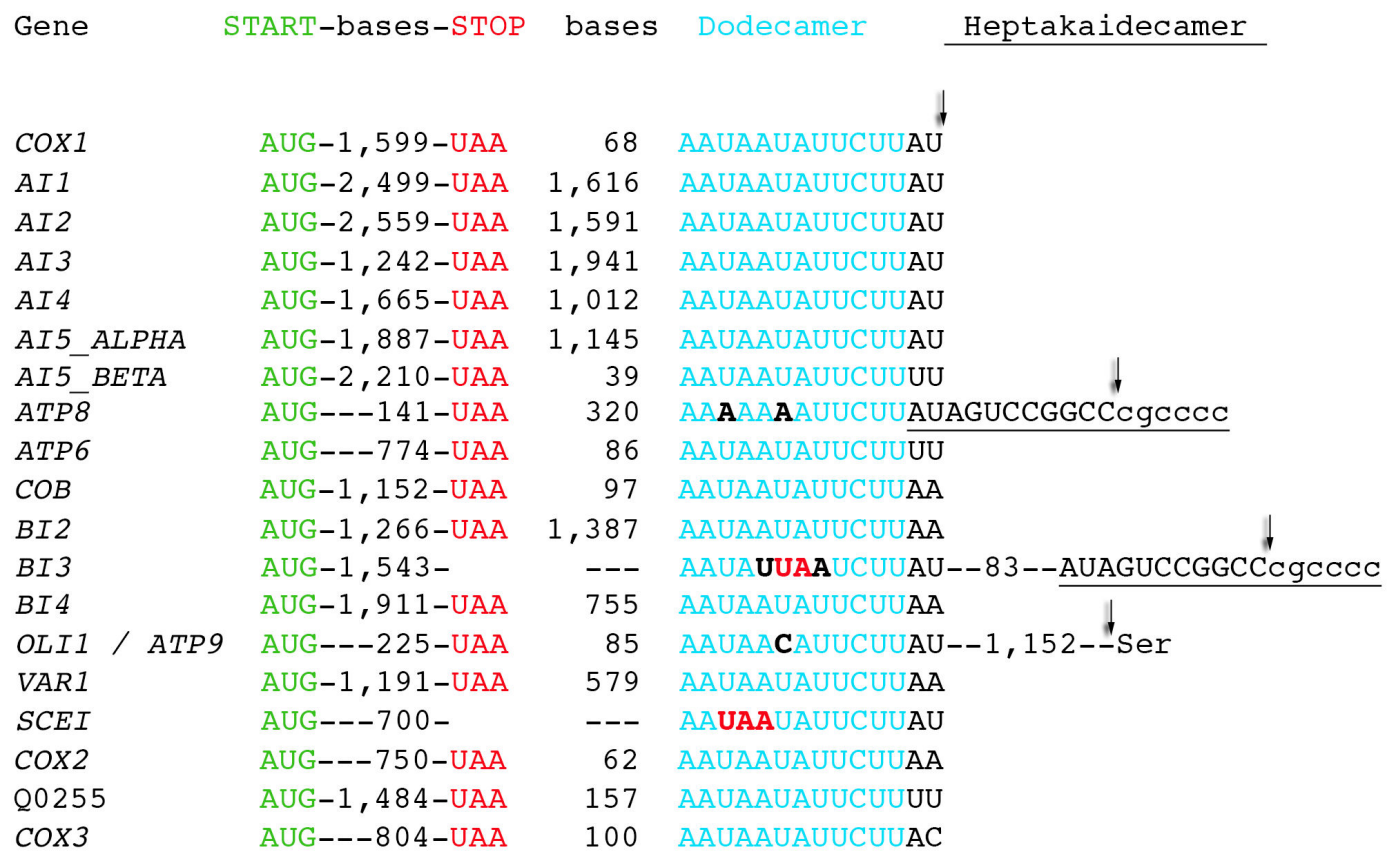
The polypeptide-coding genes of yeast S288C mitochondria. Each gene is labeled using the standard name (www.yeastgenome.org) and convention (all capital, italic, no spaces), or the systematic name (all capital, regular) if no standard name is available. Each open reading frame begins with the AUG codon (green) and ends with the UAA stop codon (red). The size of the ORF in between the start and stop codons is depicted as number of bases, as is the distance in between the stop codon and the dodecamer (12 mer) sequence, which is blue with deviations bold/black. The likely cleavage sites are depicted with a down arrow. The *BI3* and *SCEI* stop codons (bold/red) are within the dodecamer. The heptakaidecamer (17 mer) downstream of the *ATP8* and *BI3* dodecamer is underlined. The distance in between the *OLI1 / ATP9* dodecamer and the downstream tRNA (Ser) that cleaves the primary transcript via normal tRNA processing is depicted as number of bases.

In human and yeast mitochondria, AUA codes for methionine[49,50] and can be utilized as an initiator codon[51]. In yeast, only the aI5β intron-encoded maturase is thought to initiate with an AUA codon[52]. All other yeast intron-encoded proteins are in-frame with their upstream exon and thus utilize AUG as the initiation codon. Strikingly, our RNAseq data shows that alternative splicing—the first identified in yeast mitochondria—puts aI5β in-frame with the upstream exon; thus all yeast mitochondrial proteins initiate with the AUG codon (Figure 3A). We confirmed this result by specifically amplifying the splice junction by RT-PCR followed by sequencing the product (Figure 3B). Additionally, this study confirms that all yeast mitochondrial ORFs utilize the UAA stop codon (Figure 4). Both the mechanism of mitochondrial alternative splicing, and the biological advantage of using a single stop codon, remain to be determined.

### 3’ RNA ends, Dodecamer, Heptakaidecamer, and Endonucleolytic Cleavage

It has been postulated that all yeast mitochondrial ORFs except *ATP8* and *ATP9* are processed at a dodecamer sequence in the 3’ untranslated region (UTR)[53,54]. The dodecamer directs endonucleolytic cleavage of the primary mRNA and protects the mature 3’ end from exonucleolytic decay. We have determined that *ATP8* and *ATP9* both have non-canonical dodecamer sequences, suggesting an alternative mechanism of mRNA cleavage (Figure 4). In this regard, *ATP9* has a tRNA downstream of its dodecamer, and may undergo tRNA “punctuation,” that is, a mechanism of endonucleolytic cleavage of the 3’ end of primary transcripts in human and yeast mitochondria[55]. We have also discovered a 17-base heptakaidecamer RNA cleavage site immediately downstream of the *ATP8* dodecamer (Figure 4). Thus, non-canonical dodecamers may still provide protection to *ATP8* and *ATP9* mRNA 3’ ends, while cleavage has moved distally. In support of this, we also found a heptakaidecamer downstream of a deviant dodecamer at the end of the *BI3* ORF (Figure 4). 

**Figure 5:**
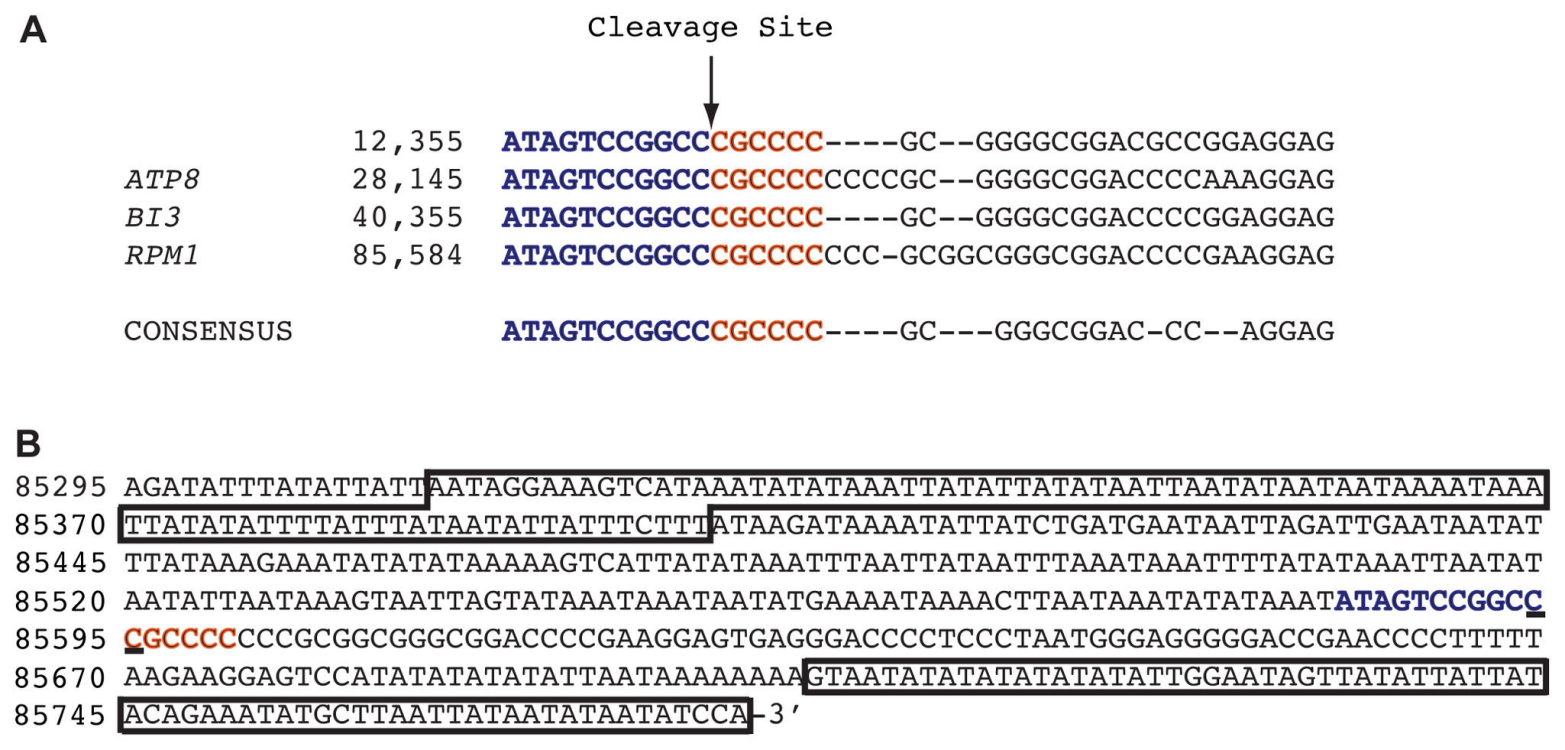
Heptakaidecamer cleavage site. (A) Alignment of four heptakaidecamer sequences. The down arrow that separates the blue and the orange sequences indicates the cleavage site. (B) The non-template strand of the 483-bp, unprocessed, RNA subunit of RNase P, derived from the mitochondrial-encoded *RPM1* gene. Boxes represent the three fragments identified in purified RNase P. Fragment 1 (89 nt) is at the 5’ end of the primary transcript, and fragments 2 (71 nt) and #3 (52 nt) overlap and are positioned at the 3’ end. The 5’ fragment is separated from the 3’ fragments by a heptakaidecamer cleavage site highlighted in blue and orange. The base immediately upstream and downstream of cleavage is underlined.

Heptakaidecamer cleavage sites are particularly easy to identify in our RNAseq data because numerous reads end precisely at the 5’ cleavage nucleotide, and numerous other reads begin precisely at the 3’ cleavage nucleotide. The heptakaidecamer is found 18 times in the S288C mitochondrial genome, but in our RNAseq dataset we only find reads associated with the RNA ends of four of them (Figure 5A). 

In addition to the two aforementioned heptakaidecamer sequences, we find a cleavage site ~3kb downstream of the end of the *15S rRNA* / Trp transcript, and a fourth cleavage site within the *RPM1* transcript (Figure 5B). This discovery is significant because mature yeast mitochondrial RNase P contains three fragments of *RPM1* but no full-length *RPM1*[56]. The heptakaidecamer separates the fragments from the primary transcript. Yeast RNase P is thus analogous to the cytoplasmic ribosome in that both are built from primary RNAs that are cleaved and processed into smaller RNAs that then assemble with proteins to operate on RNA substrates. 

### Dis3p is a Mitochondrial Protein

Mitochondrial RNA decay—first identified in yeast as the biological function of the Dss1p-Suv3p degradosome[13]—is a regulatory mechanism of mitochondrial gene expression. Humans share the Suv3p helicase[15] but lack a recognizable Dss1p homolog. The human RNase partner is PNPase[14,57], although the degradosome may not be the sole RNA decay machinery in yeast or human cells. Perhaps mitochondrial RNases have evaded detection due to redundancy, null lethality, transient localization, minute accumulation, or eclipsed distribution[58].

To identify elusive mitochondrial RNases, we scored every putative yeast RNase for null lethality, localization, and percent likelihood of mitochondrial targeting (Table 4, 5, 6, 7). Dis3p emerged as a strong candidate for a conserved mitochondrial RNase. Dis3p, an exo- and endo-RNase component of the exosome, is responsible for numerous RNA metabolic functions in the nucleus and cytoplasm[59]. In support of the bioinformatic findings, four independent proteomic studies found Dis3p in highly purified mitochondria[60-63] (Table 8). One of these studies identified a Dis3p N-terminal peptide that was processed at a canonical matrix-cleavage site[63]. Further support for Dis3p being mitochondrial comes from proteomic screens, where Dis3p co-purifies with Mas1p, the matrix protease that cleaves the N-terminal MTS from imported proteins[64,65] (Table 8). Notably, although Dis3p stably interacts with the 9-subunit exosome core, no core exosome subunits were identified in the mitochondrial proteome[60-63]. 

**Table 4:** Yeast dual exo/endo-ribonucleases.

**Standard Name**	**Systematic Name**	**Alias**	**Family**	**Reported Localization**	**Description**	**Null**	**Human Homolog**	**MitoProtII score (cs)**
*DIS3*	YOL021C	*RRP4 4MTR17*	E.coli RNase R of RNase II PIN	Nuclear Cytoplasmic Mitochondrial	Exosome RNA decay and processing	Lethal	DIS3 DIS3L DIS3L2	96.3 (30)
*NUC1*	YJL208C			Nuclear Mitochondrial	Apoptosis	Viable	EndoG	7.4 (NP)

Cleavage site nucleotide (cs).

**Table 5:** Yeast 5’-to-3’ exoribonucleases.

**Standard Name**	**Systematic Name**	**Alias**	**Family**	**Reported Localization**	**Description**	**Null**	**Human Homolog**	**MitoProtII score (cs)**
*PET127*	YOR017W			Mitochondrial	May not have RNase activity Mitochondrial RNA 5’-end processing	Viable	None	98.0 (48)
*RAT1*	YOR048C	*HKE1 TAP1 XRN2*		Nuclear Mitochondrial	Nuclear, single-stranded RNase rRNA and snRNA processing poly (A+) dependent and independent mRNA transcription termination	Lethal	XRN2	94.4 (18)
*RRP17*	YDR412W			Cytoplasmic	rRNA processing	Lethal	NOL12/NOP25	84.5 (NP)
*XRN1*	YGL173C	*KEM1 DST2 RAR5 SEP1 SKI1*		Cytoplasmic	Cytoplasmic processing (P) bodies involved in mRNA decay	Viable	XRN1	56.9 (18)

Cleavage site nucleotide (cs).

**Table 6:** Yeast 3’-to-5’ exoribonucleases.

**Standard Name**	**Systematic Name**	**Alias**	**Family**	**Reported Localization**	**Description**	**Null**	**Human Homolog**	**MitoProtII score (cs)**
*DSS1*	YMR287C	*MSU1*	RNase II	Mitochondrial	Degradosome	Viable	None	99.7 (35)
*REX2*	YLR059C	*YNT20*	RNase D	Mitochondrial	Mitochondrial genome maintenance snRNA 3’-end processing	Viable	REXO2	99.1 (33)
*REX4*	YOL080C			Nuclear	pre-rRNA processing	Viable	None	96.6 (55)
*YME2*	YMR302C	*RNA12 PRP12*		Mitochondrial	Mitochondrial genome maintenance	Viable	None	93.3 (45)
*RRP6*	YOR001W		DEDD	RNase D	Nuclear nuclear exosome	Viable	EXOSC10	35.4 (NP)
*REX3*	YLR107W		RNase D	Nuclear Cytoplasmic	3’-end processing of ncRNA, rRNA, snRNA	Viable	None	30.6 (NP)
*PAN3*	YKL025C			Cytoplasmic	Pan2p-Pan3p deadenylation	Viable		21.2 (NP)
*NGL3*	YML118W		Exo- Endo-Phosphatase (EEP)	Unknown	specific for poly-A RNAs	Viable	None	17.1 (NP)
*POP2*	YNR052C	*CAF1*	DEDD	Cytoplasmic	3’ to 5’ mRNA deadenylation Ccr4-NOT complex	Viable	CNOT7	10.5 (19)
*RNH70*	YGR276C	*REX1 RNA82*	RNase H RNase D	Nuclear	maturation of 3’ ends of 5S rRNA and tRNA-Arg3	Viable	ZFP42	3.7 (NP)
*PAN2*	YGL094C	*ECM35*		Cytoplasmic	Pan2p-Pan3p deadenylation	Viable	hPAN2	2.3 (NP)
*CCR4*	YAL021C	*FUN27 NUT21*	Exo- Endo-Phosphatase (EEP)	Cytoplasmic	3’ to 5’ mRNA deadenylation Ccr4-NOT complex	Viable	CNOT6	0.0 (NP)

Cleavage site nucleotide (cs).

**Table 7:** Yeast endoribonucleases.

**Standard Name**	**Systematic Name**	**Alias**	**Family**	**Reported Localization**	**Description**	**Null**	**Human Homolog**	**MitoProtII score (cs)**
*IRE1*	YHR079C	*ERN1*	RNase L	Nuclear	HAC1 mRNA splicing	Viable	ERN1	97.6 (45)
*NGL1*	YOL042W		Exo- endo-Phosphatase (EEP)	Mitochondrial	5.8S rRNA processing at site E	Viable	LRRC4C	96.3 (24)
*DBR1*	YKL149C	*PRP26*		Nuclear	Lariate Debranching	Viable	DBR1	40.1 (NP)
*NOB1*	YOR056C		PINc domain	Nuclear Cytoplasmic	rRNA processing	Lethal	ART 4	31.9 (NP)
*NMD4*	YLR363C		PINc domain	Cytoplasmic	No RNase Activity Shown	Viable	None	20.5 (NP)
*RNY1*	YPL123C		T2 RNase	Cytoplasmic Vacuolar	cleaves tRNA	Viable	None	17.6 (NP)
*SEN34*	YAR008W	*FUN4*		Cytoplasmic-OMM (Outer MitoMem)	tRNA 3’ splice site cleavage	Lethal	TSEN34 LENG5	4.1 (NP)
*DOM34*	YNL001W			Cytoplasmic	No-Go mRNA Decay	Viable	None	3.9 (NP)
*NGL2*	YMR285C		Exo- endo-Phosphatase (EEP)	Nuclear	5.8S rRNA processing	Viable	None	3.4 (NP)
*SEN2*	YLR105C			Cytoplasmic-OMM (Outer MitoMem)	tRNA 5' splice site cleavage	Lethal	TSEN2	3.3 (NP)
*SWT1*	YOR166C		PIN domain	Nuclear Cytoplasmic	perinuclear mRNP quality control	Viable	None	3.2 (NP)
*TRZ1*	YKR079C		tRNase Z metallo-β-lactamase	Nuclear Cytoplasmic Mitochondrial	tRNA 3'-end processing endonuclease	Lethal	ELAC2	2.7 (NP)
*RNT1*	YMR239C		RNAase III	Nuclear	rRNA, snRNA processing	Viable	None	1.3 (NP)
*YSH1*	YLR277C	*BRR5*	metallo-β-lactamase	Nuclear	3' processing of mRNAs	Lethal	CPSF2	0.7 (NP)
*RPR1*				Nuclear	ncRNA nuclear RNase P; cleaves 5' ends of tRNA precursors	Lethal	RPPH1 H1 RNA	
*RPM1*		*9S RNA*		Mitochondrial	ncRNA mitochondrial RNase P, removes 5' extensions from mitochondrial tRNA precursors	Viable	None Protein-only	
*NME1*		*RRP2*		Nuclear Cytoplasmic	snoRNA RNase MRP, cleaves pre-rRNA	Lethal	RMRP	
Group I introns			Self-Splicing	Mitochondrial	RNA component of Mito Introns	Viable		
Group II introns			Self-Splicing	Mitochondrial	RNA component of Mito Introns	Viable		

Cleavage site nucleotide (cs).

**Table 8:** Public databases that have identified Dis3p as a mitochondrial protein.

**Year**	**Journal**	**Authors**	**Results**	**Growth Conditions**
2009	Cell	Vogtle et al	N-terminal sequencing. Dis3p cleaved pre-sequence is 26aa long, ends with R, and the first amino acid of the mature protein is S (processed mitochondrial proteins typically cleave between R and S).	Sucrose or YPG
2006	Journal of Proteomic Research	Reinders et al	Dis3p peptide fingerprint	YPG
2006	Nature	Gavin et al	Mas1p Physical Interaction	YPD
2004	PLOS Biology	Prokisch et al	Dis3p peptide fingerprint	SCD or YPD
2003	PNAS	Sickmann et al	Dis3p peptide fingerprint	YPG
2002	Nature	Gavin et al	Mas1p Physical Interaction	YPD

**Figure 6:**
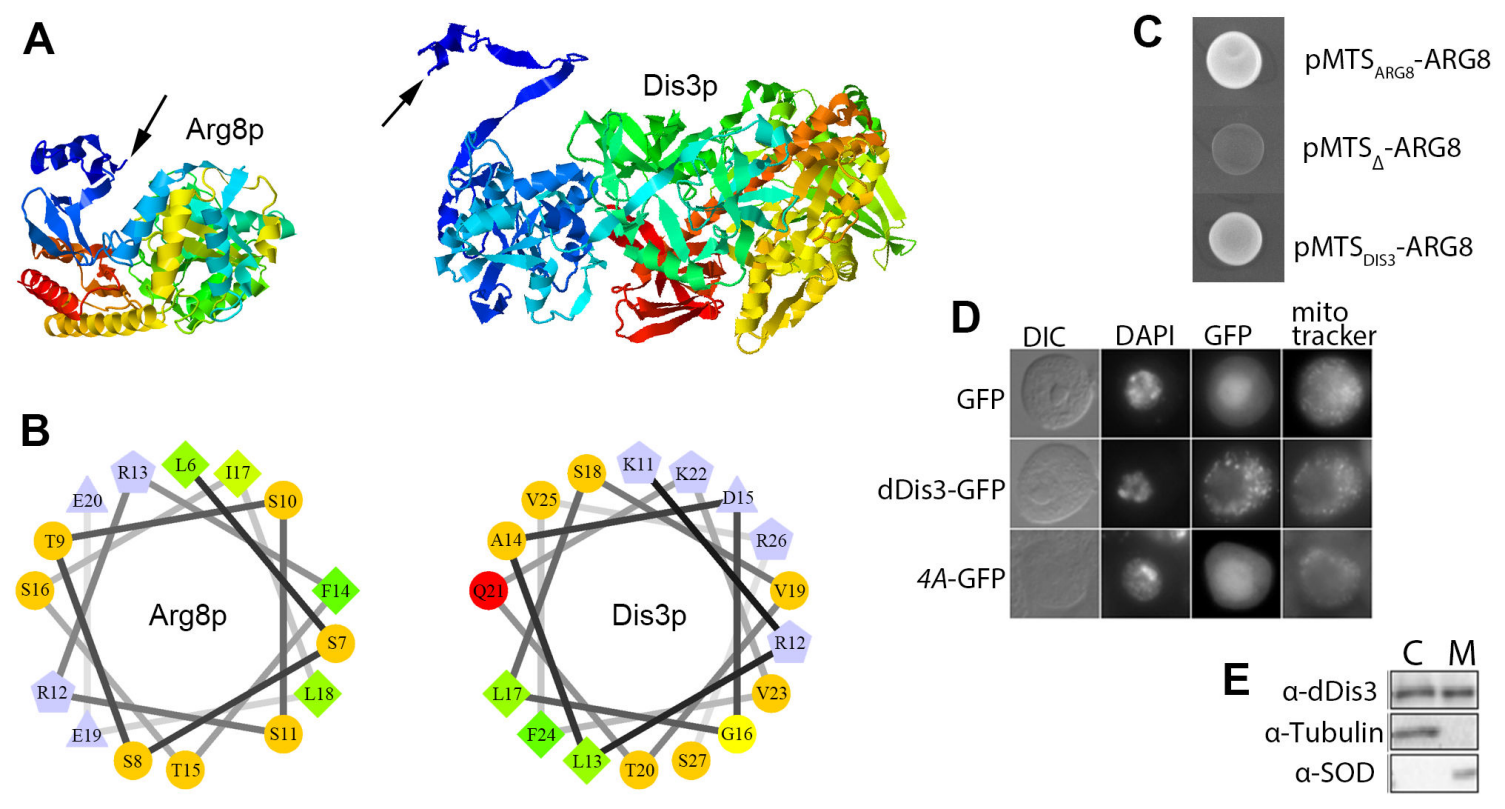
Dis3p is a conserved mitochondrial protein. (A) I-TASSER models of Dis3p and Arg8p. N-termini indicated by arrows. (B) Amphipathic alpha helix located at the N-terminus of Dis3p and Arg8p. Single letter amino acid code and position in the primary structure is indicated. (C) Growth of yeast on arginine-free medium when the MTS of *DIS3* replaces the plasmid borne MTS of *ARG8*. (D) The Drosophila dDis3 N-terminus can function as an MTS for GFP, but mutation of the MTS (4A) cannot. (E) Western blot of cell fractionation. C (cytoplasm), M (mitochondria).

To determine whether the Dis3p N-terminus is part of the tertiary structure or available to function as a signal peptide, we used I-TASSER[30,31] to comparatively model the protein structure of Dis3p to that of Arg8p, a mitochondrial matrix protein required for arginine biosynthesis. Dis3p crystal structures are available, but an ordered N-terminus has only been reported in the context of the exosome, and in this case it forms a β–hairpin when wedged between Rrp41-Rrp42 [66,67]. We chose Arg8p to use as a comparative model because this would also serve to inform our design of a reporter system (see next paragraph). The models indicate that both proteins have an available N-terminal peptide (Figure 6A). Helical wheel analysis of Dis3p and Arg8p show that each N-terminal peptide forms an amphipathic alpha helix, the basic structure of most mitochondrial import signals (Figure 6B).

**Figure 7:**
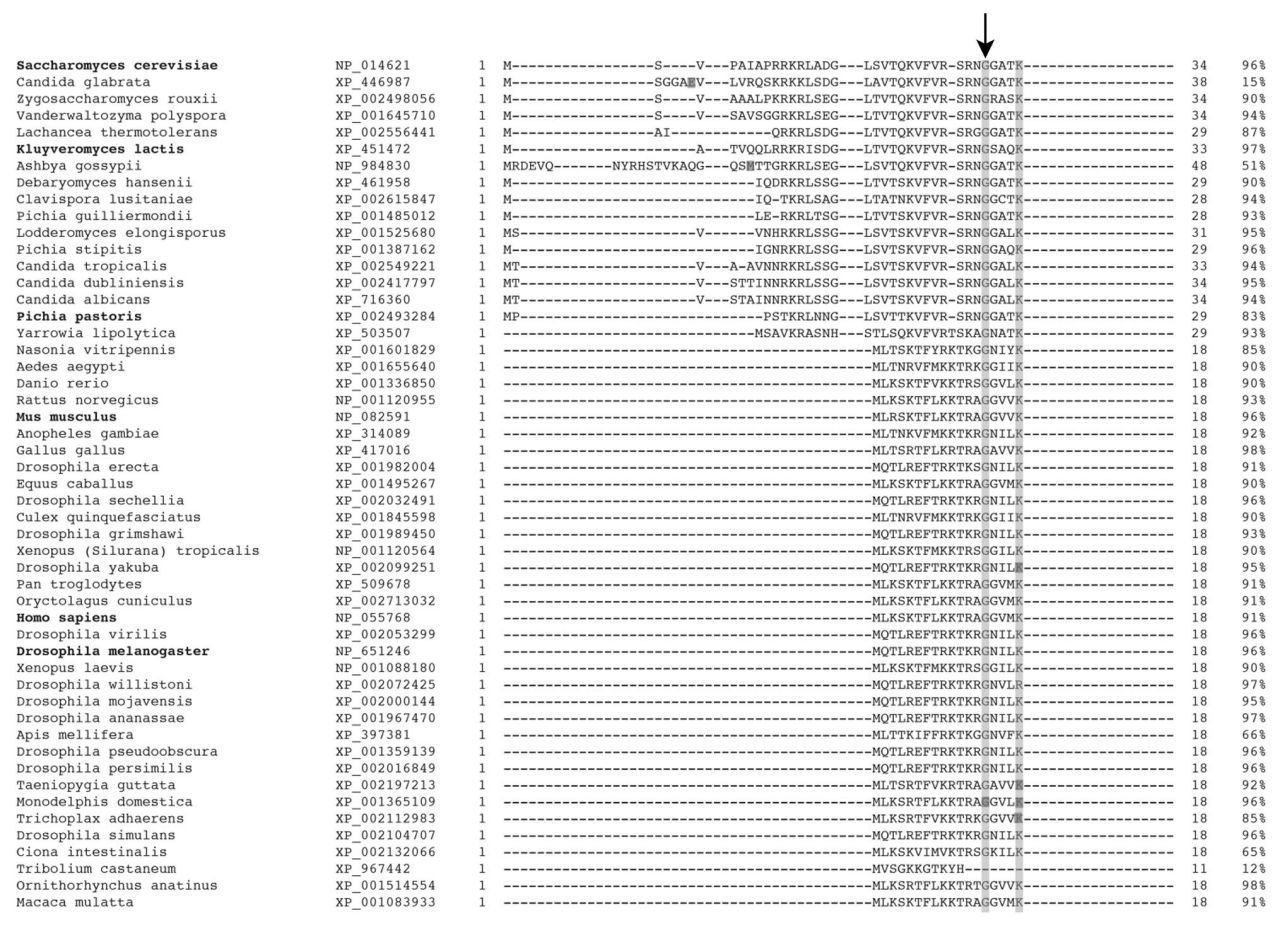
Top 50 alignments to yeast Dis3p shows a strongly conserved N-terminus. Species name is followed by NCBI reference, the amino acid sequence of the N-terminus, and the percent likelihood of mitochondrial targeting. Down arrow marks the conserved mitochondrial cleavage site.

To assess protein import to the mitochondrial matrix *in vivo*, we created a reporter system in which plasmid-borne *ARG8* rescues loss of arginine-prototrophic growth of an *ARG8*-deletion strain. Removal of the *ARG8* plasmid-borne MTS eliminates the growth rescue, whereas replacement with the *DIS3* MTS restores growth (Figure 6C). To assess conservation of Dis3p mitochondrial localization, we appended the *DIS3* MTS from *Drosophila melanogaster* to *GFP* followed by expression in fly S2 cell culture. Fluorescence is detected in mitochondria and lost when *DIS3* MTS is absent or mutagenized (Figure 6D). Cell fractionation also shows native Dis3p in S2 mitochondria (Figure 6E). Bioinformatic alignment of 50 Dis3p homologs confirms the conservation of the N-terminus (Figure 7). Thus Dis3p appears to be a genuine mitochondrial protein.

### DIS3 is Involved in Mitochondrial RNA Decay

**Figure 8:**
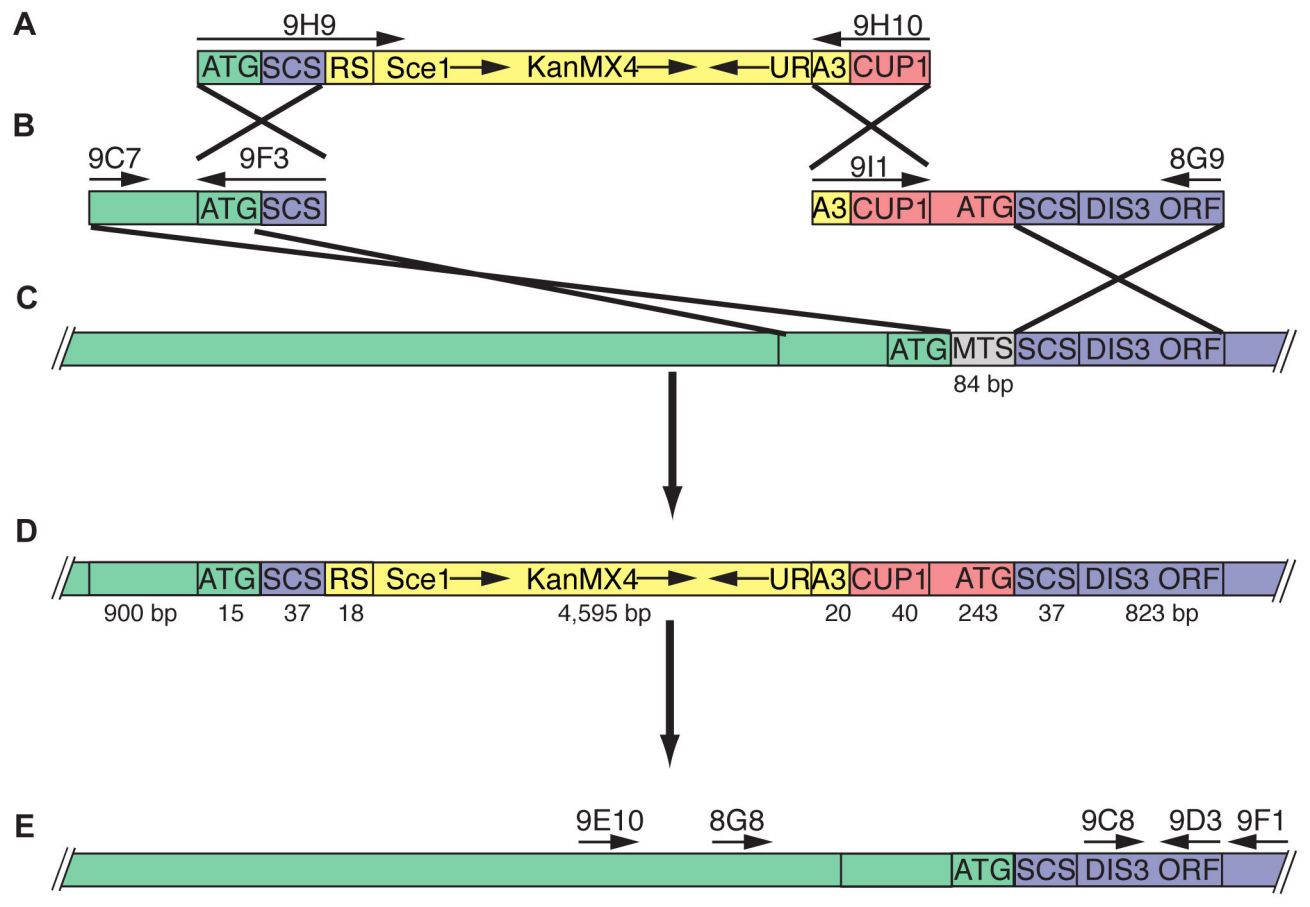
Engineering scheme to create *dis3∆mts*. (A) Amplified the CORE molecule by PCR from plasmid pGSKU using primers 9H9 / 9H10, which include targeting sequences at their 5‘ ends. (B) Amplified the upstream targeting molecule by PCR from yeast chromosomal DNA, strain BY4741, using primers 9C7 / 9F3. Amplified the downstream targeting molecule by PCR from yeast chromosomal DNA, strain *P*_*CUP1*_ *-DIS3* which is a derivative of BY4741, using primers 9I1 / 8G9. (C) Simultaneously transformed the three molecules into BY4741. (D) Transformation resulted in a strain in which the MTS is deleted, *DIS3* is expressed by the *CUP1* promoter, and identical self-cloning sites (SCS) flank the cassette. (E) Over-night growth in galactose media induced expression of *SCE1* endonuclease, which cleaved the DNA at the restriction site (RS), and the cut was repaired by homologous recombination between the SCS, which removed the cassette. The entire gene was amplified by PCR using primers 9E10 / 9F1 and sequenced with the PCR primers and internal primers 8G8, 9C8, and 9D3.

To determine the biological significance of the MTS, we engineered an allele in which *DIS3* codons 2-29 were deleted from the yeast genome (Figure 8). *DIS3* is an essential gene and thus cannot be deleted unless compensated by plasmid-borne *DIS3*[68]. Changing the genomic context could alter endogenous expression so rather than use a plasmid-based system; we created a chromosomal mutant of *DIS3* in which only the MTS is deleted. The protein resulting from the *dis3∆mts* allele is expected to be mitochondrial excluded. Yeast strains harboring the *dis3∆mts* allele are not growth impaired on fermentable or non-fermentable carbon sources, suggesting a redundant function. This is consistent with truncation experiments in *S. pombe* in which the N-terminal 74 amino acids of Dis3p were determined not to be essential[69]. To investigate the contribution of Dis3p to mitochondrial RNA metabolism, we performed replicate RNAseq analyses on RNA extracted from purified *DIS3* and *dis3∆mts* mitochondria. For *DIS3* mitochondria, we found 71% coverage of the positive strand and 25% coverage of the negative strand. We also found a 9% increase in the base coverage for both the positive and negative strand in *dis3∆mts*, suggesting that Dis3p has a role in modulating the abundance of RNAs that would otherwise be too unstable to detect, much like the role of the exosome in nuclear RNA surveillance[70]. This coverage contrasts with the human mitochondrial transcriptome, which has ~98% of both strands transcribed[11]. Whereas in that study, ~1.4 million uniquely mapping reads provided saturation of coverage, we obtained ~36 million uniquely mapping reads.

**Figure 9:**
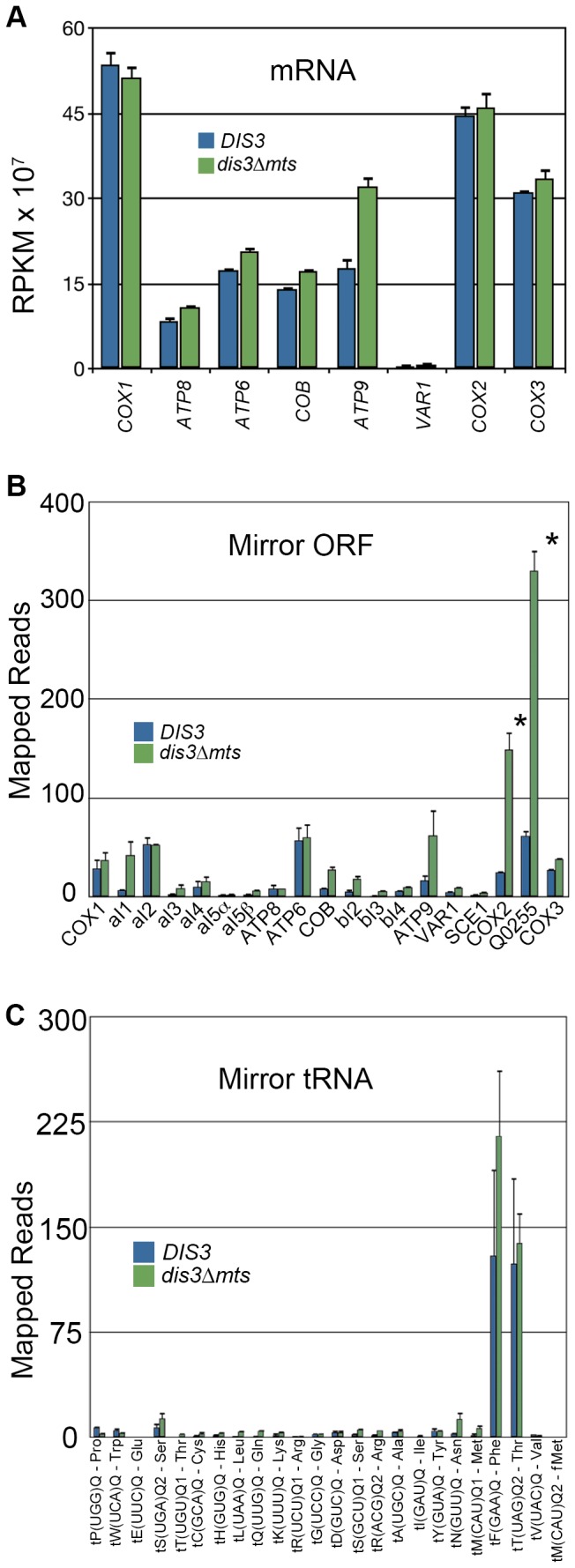
The mRNA and mirror-RNA profiles in *DIS3* and *dis3∆mts*. Wild type (DIS3) reads are first / blue and the mutant (dis3∆mts) are second / green. Error bars = s.e.m. Asterisk denotes a statistically significant difference with a p-value less than 0.01 as determined by the DESeq statistical method[29]. (A) Read count in RPKM for the eight mRNAs that encode proteins required for oxidative phosphorylation. (B) Total number of reads that map to each mirror ORF. (C) Total number of reads that map to each mirror tRNA.

**Figure 10:**
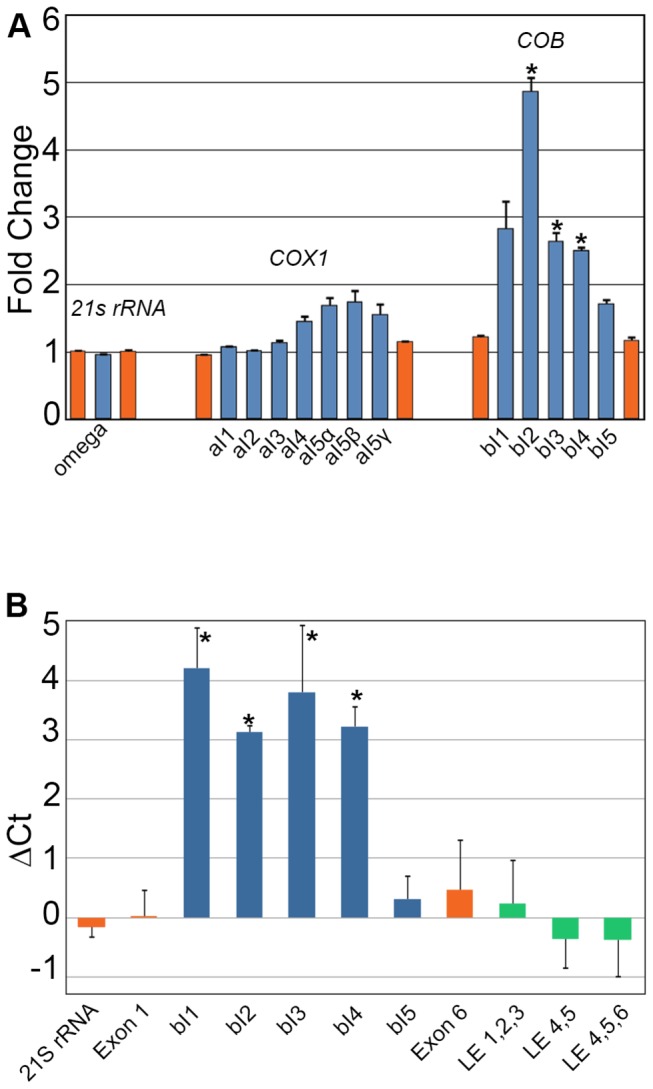
Hyper-accumulation of Group I and Group II intron RNA in *dis3∆mts*. (A) Fold change of the RPKM value between *DIS3* and *dis3∆mts* for the exons and introns of *21S* *rRNA*, *COX1*, and *COB*. Values >1 indicate an increase in reads in *dis3∆mts*. Only the first and last exon (orange) of each intron-containing gene is shown for purposes of clarity. Error bars = s.e.m. Asterisk denotes a statistically significant difference with a p-value less than 0.01 as determined by the DESeq statistical method[29]. (B) RT-qPCR of *DIS3* and *dis3∆mts* RNA. Change in Ct value for the first exon of *21S* *rRNA* (orange), the first and last exon of *COB* (orange), the five *COB* introns (blue), and ligated exons (LE / green) of the *COB* gene. Values greater than zero indicate greater abundance in *dis3∆mts*. Error bars = s.e.m. Asterisk denotes a statistically significant difference with a p-value less than 0.01 as determined by the Student’s t-test.

There is no difference in steady-state levels of mRNAs, with the exception of *ATP9*, which is ~2-fold increased in *dis3∆mts* (Figure 9A). We find that most tRNA and mRNA genes have corresponding antisense transcripts, which are analogous to so-called “mirror” RNA in human mitochondria[15]. In the *dis3∆mts* mitochondria, notably, some of these RNAs accumulate (Figure 9B,C). We also found a striking increase in the accumulation of some group intron RNAs of the *COB* gene when Dis3p is mitochondrial excluded (Figure 10A). We confirmed by RT-qPCR the hyper-accumulation of RNA derived from introns bI1–thru-4. Importantly, we saw no change in the abundance of *COB* ligated exons or control RNAs (Figure 10B). As the degradosome is the only known protein complex to decay mitochondrial intron RNA[71], it remains to be determined if Dis3p interacts directly with these RNAs, in concert with or independent of Suv3p and Dss1p.

## Discussion

### Bioinformatic Construction of the S288C Mitochondrial Transcriptome

We have assembled a mitochondrial transcriptome map for the yeast S288C reference strain. In this context a transcriptome map is basically a “parts list” of all the RNAs of a system, and a description of their boundaries, their physical location on the genome, and their abundance. Building a map is a step towards elucidating how all of the parts of the mitochondria act in concert to precisely balance the energy needs of the cell. This overarching goal cannot be accomplished without first characterizing the parts themselves.

To date no transcriptome map has been built for any yeast strain. We chose to use yeast S288C because of the numerous scientific resources associated with this strain. However, an enormous body of mitochondrial RNA literature has accumulated around a few yeast strains that are not isogenic to S288C. We therefore used the primary literature in conjunction with the sequenced S288C mitochondrial genome to build a parsimonious transcriptome map. This provides a model for the iterative work of testing and refinement.

 We modeled the identity and 5’ boundary of all primary transcripts by identifying the minimal number of promoters to account for transcription of all known mitochondrial genes and active *ori*. Although twenty-eight potential yeast mitochondrial promoters, centered on the nonanucleotide sequence, have been reported [72,73], we only used the minimal number of promoters. This was done to avoid the inclusion of promoters that are reported to be active *in vitro* [73-77] or in another yeast strain[44,45,78-80], but which may not be active in S288C. 

In addition to setting the minimal number of transcripts to 14, our results also expand the established size of the promoter from 9 to 20 base pairs. This is significant because modulation of transcription initiation is a known response to changes in mitochondrial function[77]. The initiating nucleotide of the yeast mitochondrial promoter is always adenosine *in vivo*, despite observed activity when changed to any other nucleotide *in vitro*[74]. The use of ATP to make the first nucleotide of the transcript has likely been evolutionarily conserved because it provides a benefit to the cell as an ATP sensor[77]. Our bioinformatic analysis has increased the sequence space that is potentially part of the regulatory apparatus, and, due to the conservation we see, there are likely to be other parts of the promoter that provide a cellular benefit.

We modeled the 3’ end of each primary transcript by searching the mitochondrial genomic sequence for dodecamer or tRNA sequences because there is no known transcriptional terminator or polyadenylation in yeast mitochondria[13]. The RNA polymerase transcribes beyond the dodecamers and terminal tRNAs and is released by an unknown mechanism. The 3’ end is formed by cleavage 2 bp downstream of the dodecamer in the case of ORFs[54] or via endonucleolytic processing of a tRNA. The presence of the dodecamer likely provides protection from decay for mRNA, while the rRNA and tRNA are protected by their secondary structures, but the stabilizing mechanism for these RNAs is unknown. 

A complete transcriptome map includes the *ori* because an RNA that is derived *de novo* from a nonanucleotide-containing promoter primes DNA synthesis[81,82]. To our knowledge, *ori2*, *3*, and *5* are the only oris that have been shown to be active in every report in which they have been tested. The other five oris have been shown to be inactive or only active in some strains[83]. We thus built a model of the S288C mitochondrial *ori* based on *ori2*, *3*, and *5*. 

We found the consensus *ori* to fit nicely the current model[84], which begins with a promoter followed by a G-rich sequence, an AT spacer, and then GC-AT-GC rich sequences that likely fold into a hairpin. The G-rich sequence is 4 bp downstream of the 20-bp consensus promoter for *ori2*, but overlaps with the last 4 bp of the *ori3* and *ori5* promoters. Thus, we find the spacing between the end of the promoter and the start of the G-rich sequence to have some flexibility. We compared the promoters of the other five *ori* and found that each has an insert that would likely disrupt transcription initiation, and, hence, render the *ori* inactive. The mechanism by which the same promoter is used for the dual roles of DNA synthesis and regulated transcription initiation remains to be identified, but may involve cis-acting elements outside of the promoter[85].

### RNAseq Analysis of the S288C Mitochondrial Transcriptome

The primary purpose of our RNAseq analysis was to quantify RNA abundance, and, as such, we did not optimize for the detection of transcription start sites (TSS), RNA 3’ ends, or active *ori*. Such optimization would have required rRNA and tRNA subtraction to get deep coverage of TSS, which in some cases are unstable. For example, those TSS that are followed by a tRNA are undetectable in our RNAseq data. This is likely due to the fact that tRNA are removed from their primary transcripts by endonucleolytic cleavage, and there is no known stabilizing sequence between the TSS and the tRNA cleavage site. Thus the freed 5’ RNA would likely be a strong substrate for RNA decay. We also did not attempt to detect RNA-DNA hybrid molecules, which would be necessary to authenticate the active *ori*. Our RNAseq data is consistent with our model of TSS, RNA 3’ ends, and *ori*, but further experimental validation is required**.**

We precisely mapped the 3’ terminal nucleotide of all 24 mitochondrial-encoded tRNA due to the presence of non-encoded CCA trinucleotide. Simultaneously identifying the boundaries and measuring the quantities of all tRNA should provide a valuable tool to investigate tRNA metabolic pathways. Mitochondrial tRNA is processed by endonucleolytic cleavage at the 5’ and 3’ end by RNase P and RNase Z respectively, but most molecular components of the processing, modifying, surveillance, stability, and decay complexes have not been identified[86].

Yeast mitochondrial RNA abundance has been measured numerous times, but with various strains, techniques and standards, and only on a limited number of RNAs[87]. We choose an RNAseq approach because it allows us to simultaneously measure the abundance of all mRNA, rRNA, tRNA, and other ncRNA. This gives us the ability to refine our transcriptome map, and allows us to identify proteins that are involved in mitochondrial RNA metabolism by comparing all RNAs in mutant to wild-type yeast. This prevents the exclusion of proteins that only work on a subset of RNAs. 

The utility of measuring all RNAs simultaneously can be seen in the example of Var1p, a ribosomal protein that was recently shown to be a negative regulator of mitochondrial DNA maintenance[88]. During conditions of enhanced mitochondrial need the cell could potentially benefit from negative regulation of Var1p activity. When yeast is grown in conditions that require enhanced mitochondrial activity, we find that *VAR1* has the lowest RNA abundance of any mitochondrial gene (Figure 9A). It is tempting to speculate that one mechanism of regulating Var1p activity is through modulating protein abundance via mRNA abundance. 

Previous studies have suggested that differential yeast mitochondrial RNA abundance depends upon rates of transcription initiation, transcription termination/attenuation, RNA processing, and RNA decay[89]. *VAR1* is co-transcribed with *ATP9*, but opposed to Var1p, enhanced Atp9p activity would be valuable to the cell during a time of enhanced mitochondrial activity. In fact, *ATP9* is ~20-fold greater in RNA abundance over *VAR1* in the conditions we tested. Further work will be required to determine if transcription is attenuated after the RNA polymerase passes the *ATP9* ORF—as is the case for the co-transcribed *Glu* - *COB* genes[90]—or if the difference in RNA abundance is due to a post-transcriptional mechanism. We conclude that our transcriptome map is a valuable tool for elucidating the interconnected processes of genome maintenance and gene expression.

### Dis3p an Active Participant in Mitochondrial Intron Decay?

Dis3p associates with a 9-subunit core ring structure (Exo-9) in the cytoplasm of *S. cerevisiae* to form an exosome that contributes to the processing and turnover of numerous cellular RNAs[68,91,92]. Dis3p is the only component of the cytoplasmic exosome with RNase activity, being a 3’-5’ hydrolytic exonuclease[93,94] and having a PIN domain required for endonucleolytic activity[59,95-97]. Dis3p and Exo-9 are also present in the nucleus, where they associate with the Rrp6 RNase to form an exosome involved in RNA surveillance[98-101]. 

Our bioinformatic analysis of all yeast RNases identified Dis3p as a candidate for mitochondrial localization. Although numerous proteomic studies show Dis3p in mitochondria, the same studies do not find other components of the exosome within the mitochondria[60-65]. There is precedence for Dis3p acting independent of the exosome[97,101-103]. For example, Dis3p can bind and degrade RNA *in vitro* in the absence of any other proteins[22,93,94,97,104]. Thus Dis3p may be a mitochondrial RNase that acts independent of the core. 

RNAseq analysis of the *dis3∆mts* strain shows significantly more bI2, 3, and 4 intron RNA than in the wild type. Due to greater variability in the results for bI1, the difference did not reach our stringent threshold of significance. However, RT-qPCR confirmed the RNAseq results and showed a significant hyper-accumulation of bI1 intron RNA. Hyper-accumulation of intron RNA can be due to enhanced transcription of the primary transcript, which could be detected as an accumulation in the amount of the first exon. Accumulation of intron RNA could also result from a defect in splicing, as un-spliced RNA is often more stable than freed intron RNA, and this can be scored as a decrease in the accumulation of ligated exons[71]. We found no difference in the abundance of exons or ligated exons and thus the hyper-accumulated introns are not a result of aberrant transcription or splicing. Hyper-accumulation of intron RNA in *dis3∆mts* is likely due to a defect in RNA decay, which is consistent with Dis3p endo- and exo-ribonuclease activity. However, this study has not addressed if Dis3p interacts directly with any of these RNAs, or if ribonuclease activity is required for these effects.

 We could find nothing in common among the four affected introns that would suggest a reason for their hyper-accumulation in the mutant, while bI5 and all other introns are largely unaffected. A somewhat similar phenomenon was found for the aI5β and bI3 introns, in that the *suv3-1* allele specifically affects the splicing of these two introns[105], due to a defect in degradosome-promoted decay of excised intron RNA[71]. These two group I introns belong to different subclasses and do not share significant sequence homology[106]. It was determined that the common factor responsible for the RNA-decay defect was Mrs1p[71], which is required for the splicing of both introns[107,108]. It is likely that Mrs1p must be removed from excised intron RNA before decay can occur, but this is blocked in the *suv3-1* background, which causes a defect in recycling of Mrs1p and hence a block in splicing[71]. Thus there may be a protein, or a structure in common with the bI1-4 RNAs, and Dis3p may act in concert with, or upstream of the degradosome to generate “degradosome-friendly” substrates via RNA binding, exonuclease, or endonuclease activities. Dis3p may also promote the decay of other RNAs, but the affect is masked in our experiments by redundancy with another protein.

It is a formal possibility that un-importable Dis3p could indirectly affect mitochondrial RNA decay by degrading cytoplasmic *SUV3* or *DSS1* transcripts, thereby depleting mitochondrial Suv3p or Dss1p. However, we consider this unlikely because the most dramatic defect of *SUV3* or *DSS1* perturbation is massive accumulation of the omega intron RNA[109]—and there is no change in the abundance of this RNA in the *dis3∆mts* strain (Figure 10A). We favor the more parsimonious explanation: yeast Dis3p directly mediates mitochondrial RNA decay similar to its roles in the nucleus and cytoplasm. This interpretation is in keeping with the current exozyme hypothesis, in which Dis3p is predicted to have some exosome-independent functions[110]. Further work will be required to determine if authentic untagged Dis3p localizes to the matrix, if Dis3p interacts directly with mitochondrial RNA, and if RNA decay depends on Dis3p ribonuclease and RNA binding activities.

## Conclusion

We present here the first yeast mitochondrial RNAseq study and the first demonstration of mitochondrial Dis3p. Molecular components of RNA processing, stability, and decay complexes still remain to be identified, and we have begun to address this by excluding individual proteins from the mitochondrion, and then measuring the abundance of all RNA species. In addition to *DIS3*, other candidates have been identified and excluding them individually and in tandem may uncover novel or redundant functions. By discovering the individual components we will be able to address the systems objective of understanding how all of the working parts of the mitochondrion coordinate to provide the energy balance that is crucial to life. 

## Supporting Information

File S1 **File includes Tables S1-S6.**
**Table S1** Yeast strains used in this study.
**Table S2** Primers used to sequence aI5β alternative-spliced junction.
**Table S3** Plasmids used in the functional assay for mitochondrial matrix localization.
**Table S4** Primers used in the functional assay for mitochondrial matrix localization.
**Table S5** Primers used in yeast fly mutagenesis experiments.
**Table S6** Primers used in RT-qPCR.
(DOCX)
