# Supplementary material for: The Mitochondrial RNA Landscape of Saccharomyces cerevisiae
Source: PLoS One. 2013 Oct 15;8(10):e78105. doi: 10.1371/journal.pone.0078105 (PMC3797045; doi:10.1371/journal.pone.0078105)
Supplement: File S1 — File includes Tables S1-S6. Table S1 Yeast strains used in this study. Table S2 Primers used to sequence aI5β alternative-spliced junction. Table S3 Plasmids used in the functional assay for mitochondrial matrix localization. Table S4 Primers used in the functional assay for mitochondrial matrix localization. Table S5 Primers used in yeast fly mutagenesis experiments. Table S6 Primers used in RT-qPCR. (DOCX) [file pone.0078105.s001.docx]

**Table S1.** Yeast strains used in this study.

| **Strain** | **Genotype** | **Source** |
| --- | --- | --- |
| BY4741 | *MATa his3-∆1 leu2-∆0 met15-∆0 ura3-∆0* | Open Biosystems |
| *dis3Δmts* | *MATa his3-∆1 leu2-∆0 met15-∆0 ura3-∆0 dis3-ΔN29* | This study |
| arg8-∆0 | *MATa his3-∆1 leu2-∆0 met15-∆0 ura3-∆0 arg8-∆0* | This study |

**Table S2.** Primers used to sequence aI5β alternative-spliced junction.

| **Primer** | **Description** | **Sequence 5’-to-3’** |
| --- | --- | --- |
| 3A5 | Reverse – 7^th^-exon - cDNA | GAAAATGTCCCACCACGTAG |
| 3F3 | Forward – 6^th^-exon – PCR & sequence | GCTCTAATCCATGGTGGTTCAATTAGATTAGCACTACC |
| 3I4 | Reverse – ORF in intron – PCR & sequence | ACCATCTCCTTCAAATAATCCAGA |

**Table S3.** Plasmids used in the functional assay for mitochondrial matrix localization.

| **Name** | **Insert description** |
| --- | --- |
| pMTS_ARG8_-ARG8 | *ARG8* gene, chromosome XV, coordinates 58548...60422 |
| pMTS_∆_-ARG8 | Same as pARG8, minus 60 base pairs (4 thru 63) of the ORF |
| pMTS_DIS3_-ARG8 | Same as pARG8, except base pairs 4 thru 63 are replaced with 4 thru 105 of the *DIS3* ORF |

The centromeric yeast shuttle vector pRS415 is used as the backbone for all plasmids.

**Table S4.** Primers used in the functional assay for mitochondrial matrix localization.

| Plasmid | Plasmid | Primer | Sequence 5’-to-3’ |
| --- | --- | --- | --- |
| pMTS_ARG8_-ARG8 | p2G04 | 9F9 | CGACGGTATCGATAAGCTTGATATCGAATTCCTGCAGCCCGGGATTCGATATGGTTGCCA |
| pMTS_ARG8_-ARG8 | p2G04 | 9G1 | TATAATCAATGTCATGATCTTTGTAGTCTCCATCATGGTCCTTGTAATCGGATCCCTTTTCCTCTAAAATGCTTGTAAATCTTCTTGATG |
| pMTS_ARG8_-ARG8 | p2G04 | 9G2 | GACCATGATGGAGACTACAAAGATCATGACATTGATTATAAAGACGACGATGATAAGGCCTTTCAAGTGACCACTTACTCTAGACCT |
| pMTS_ARG8_-ARG8 | p2G04 | 9F10 | AAGGGAACAAAAGCTGGAGCTCCACCGCGGTGGCGGCCGCTGCCAATGGTAGACACCCTC |
| pMTS_∆_-ARG8 | p2H07 | 10C10 | CGTTTGTTAGAATAATTCAAGAATCGCTACCAATCATGGGATCCGATTACAAGGACCATG |
| pMTS_∆_-ARG8 | p2H07 | 10D3 | ATTTGCATGGCCTAATGCGGTCAC |
| pMTS_DIS3_-ARG8 | p2H09 | 10C2 | CGTTTGTTAGAATAATTCAAGAATCGCTACCAATCATGTCAGTTCCCGCTATCGCCCCCA |
| pMTS_DIS3_-ARG8 | p2H09 | 10J2 | CTTTGTAGTCTCCATCATGGTCCTTGTAATCGGATCCGATCTTGGTAGCGCCTCCATTAC |

**Table S5.** Primers used in yeast and fly mutagenesis experiments.

| **Name** | **Template** | **Purpose** |
| --- | --- | --- |
| 9C7 | gDNA | In vitro synthesis of Upstream Targeting Molecule |
| TTCCTCTTGCCGTGTTGGTTGTCATCATCG | | |
| 9F3 | gDNA | In vitro synthesis of Upstream Targeting Molecule |
| TTAAATAGTGTTCTCTTACGATCTTGGTAGCGCCTCCCATGTTGTTTTGGCCTGTATGAT | | |
| 9H9 | pGSKU | In vitro synthesis of CORE Molecule |
| GGCCAAAACAACATGGGAGGCGCTACCAAGATCGTAAGAGAACACTATTTAATAGGGATAACAGGGTAATTTGGATGGACGCAAAGAAGT | | |
| 9H10 | pGSKU | In vitro synthesis of CORE Molecule |
| AGCAAAAGAATCTCTTGACAGTGACTGACAGCAAAAATGTTTCGTACGCTGCAGGTCGAC | | |
| 9I1 | gDNA | In vitro synthesis of Downstream Targeting Molecule |
| GTCGACCTGCAGCGTACGAAACATTTTTGCTGTCAGTCACTGTCAAGAGATTCTTTTGCT | | |
| 8G9 | gDNA | In vitro synthesis of Downstream Targeting Molecule |
| ACCTGGTCACCGTTGAATGCTCTA | | |
| 9E10 | gDNA | PCR Genomic Integration/Excision Site |
| CTTCAAATTACTTGTGTCGAACGAGCCG | | |
| 9F1 | gDNA | PCR Genomic Integration/Excision Site |
| TGGGTATTTATGAGTGGTGGGCCA | | |
| 9E10 | PCR | Sequence Targeting/Excision Site |
| CTTCAAATTACTTGTGTCGAACGAGCCG | | |
| 8G8 | PCR | Sequence Targeting/Excision Site |
| TTAGCGCCGTAGCTTCTTTAGGGT | | |
| 9C8 | PCR | Sequence Targeting/Excision Site |
| ACCAAGTGTCCGCAAATTGTCGTC | | |
| 9D3 | PCR | Sequence Targeting/Excision Site |
| GACGACAATTTGCGGACACTTGGT | | |
| Dis3F/1 | Mtn-Dis3 | Amplification of dDis3 N-terminus |
| GCGAGATCTAAAATGCAAACTTTAGCGGAATTTACG | | |
| Dis3R/L35 | Mtn-Dis3 | Amplification of dDis3 N-terminus for in-frame fusion with GFP |
| GCGGGATCCCAGCTCGGAGCCGCAGCCGATGTC | | |
| Dis3F/MTS4A | Mtn-Dis3 | Amplification and mutagenesis of dDis3 N-terminus |
| CGCAGATCTAAAATGCAAACTTTACGCGAATTTACGGCTGCTACTGCAGCCGGCAACATTCTGAAGATTG | | |

**Table S6.** Primers used for RT-qPCR.

| **Primer** | **Gene** | **Direction** | **Sequence 5’-to-3’** |
| --- | --- | --- | --- |
| 11E06 | 21S rRNA First Exon | Forward | AGCGAAATTCCTTGGCCTATAA |
| 11E07 | 21S rRNA First Exon | Reverse | CCGTCTTGCTGAAGGTACATAG |
| 11E08 | COB First Exon | Forward | ATGGTTCATATAGATCACCAAGAGT |
| 11E09 | COB First Exon | Reverse | TCTGTCCATAAACACAACAATAACC |
| 11E10 | bI1 | Forward | GAAAGCATGCTAACTTCAATATAGG |
| 11F01 | bI1 | Reverse | CAGAACTGTACGTACTACTTTCATAG |
| 11F02 | bI2 | Forward | ATTTATGGTTCAATATTAGGAGATGGTC |
| 11F03 | bI2 | Reverse | GTTGAAATACAATTCTTGTTCCTTTACC |
| 11H02 | bI3 | Forward | TTGGTGGTTTATTTGAAGGAGATGGTTGAATTACTAT |
| 11H03 | bI3 | Reverse | CCATCTTTTATTTTTAATTTTTTAATTGTTACTTTACCAATACCT |
| 11F06 | bI4 | Forward | AGAAGATAATCAGCAGGTAACCAA |
| 11F07 | bI4 | Reverse | GTGCGTGTAGTCTCTGAAGTATC |
| 11F08 | bI5 | Forward | GGGAACTCCCATAAGGAGTTTAAT |
| 11F09 | bI5 | Reverse | TCGCTGCAAGTTGTCATAGT |
| 11F10 | COB Last Exon | Forward | CCATTTACTGATAGAAGTGTAGTAAGAG |
| 11G01 | COB Last Exon | Reverse | GCATGCTCCAATTTGTCCTAATA |
| 11G02 | COB Ligated Exons 1-2-3 | Forward | TGGACAGATGTCACATTGAGG |
| 11G03 | COB Ligated Exons 1-2-3 | Reverse | ACCCACCTCATAATCAAGATACAA |
| 11G04 | COB Ligated Exons 4-5 | Forward | CTCAGCAATTCCATTTGTAGGTAAC |
| 11G05 | COB Ligated Exons 4-5 | Reverse | ACGCAAAGAATCTCTGGATTAGA |
| 11G06 | COB Ligated Exons 4-5-6 | Forward | ACTTTAGGTGTACCTGAATGATACT |
| 11G07 | COB Ligated Exons 4-5-6 | Reverse | GCTGCAAACATTAGAATAACTCCT |
